# Northern Light Reviews – Rethinking Plant Stress Biology in a Multifactorial World

**DOI:** 10.1111/ppl.70983

**Published:** 2026-08-05

**Authors:** Lidia S. Pascual, Rosa M. Rivero, Sara I. Zandalinas, Ron Mittler

**Affiliations:** ^1^ Department of Biology, Biochemistry and Environmental Sciences University Jaume I Castelló de la Plana Spain; ^2^ Department of Plant Nutrition Center of Edaphology and Applied Biology of Segura (CEBAS) – CSIC Murcia Spain; ^3^ The Division of Plant Sciences Technology, Interdisciplinary Plant Group, College of Agriculture, Food and Natural Resources, Christopher S. Bond Life Sciences Center University of Missouri Columbia Missouri USA; ^4^ Advanced Agriculture Initiative Tel‐Hai University of Kiryat Shamona and the Galilee Kiryat Shamona Israel

**Keywords:** climate change, hormonal crosstalk, multifactorial stress, photosynthesis, stomatal regulation, stress combination

## Abstract

The accelerated pace of climate change, soil degradation, environmental pollution, and pathogen outbreaks subjects trees and crops growing in different parts of the world to multiple abiotic/biotic stress conditions, simultaneously or sequentially. Unfortunately, most studies conducted to date, including studies of two‐stress combinations, do not mimic the complex multifactorial stress combination (MFSC) environment plants experience in nature. Here, we summarize evidence from recent MFSC studies of three or more stresses simultaneously impacting plants, and argue that environmental complexity, i.e., a high number of interacting stress factors, functions as a system‐level variable shaping plant performance. Across different model and crop plants, the progressive increase in stress complexity results in non‐additive effects that cause marked declines in growth, photosynthetic rates and yield, even when the level of each individual stress involved in such MFSC remains moderate. Multi‐omics analyses reveal that MFSC does not simply induce stress‐response pathways but redistributes regulatory control within hormonal networks, most prominently within abscisic acid signaling, accompanied by reduced physiological performance and repression of photosynthesis‐related genes. These responses scale nonlinearly with increasing complexity and are consistent with threshold‐like shifts in regulatory function, rather than simple additive effects. We further present a complexity‐driven regulatory model in which rising combinatorial load progressively narrows regulatory flexibility and promotes survival‐oriented strategies over plant growth. Finally, we discuss how recognizing stress complexity as an organizing variable could reshape breeding priorities and predictive crop modeling under increasingly complex environmental scenarios.
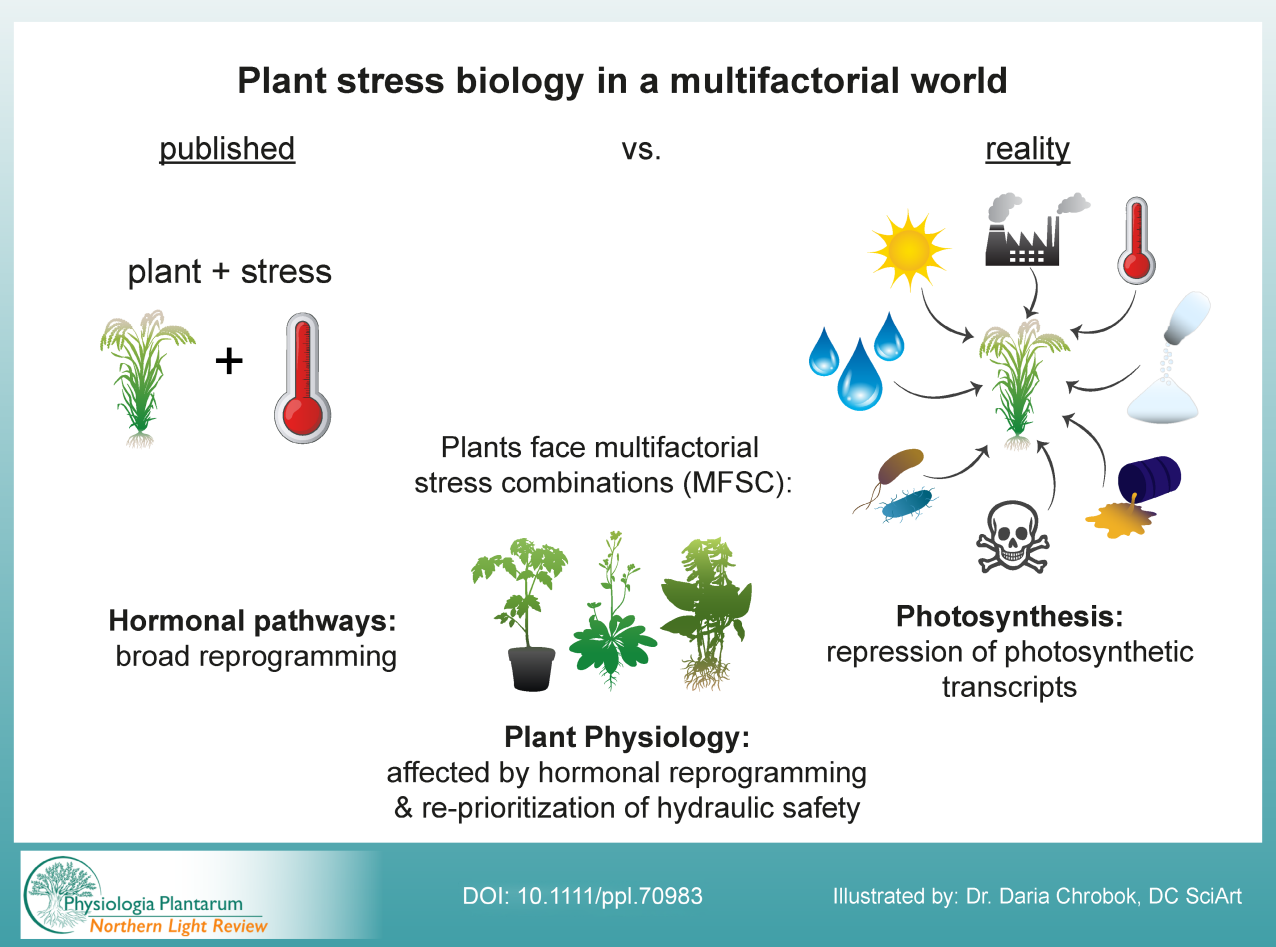

## 1 Introduction

### 1.1 Environmental Complexity as the New Baseline for Plant Stress Biology

Environmental conditions across terrestrial ecosystems are changing rapidly, with multiple environmental stresses occurring simultaneously or sequentially. Anthropogenic climate change, including global warming, more severe and frequent floods and storms, increased land use, atmospheric, soil, and water pollution, soil degradation, microbiome decline, and hydrological disruptions, are reshaping environmental landscapes at both regional and global scales (Meehl and Tebaldi 2004; Teuling 2018; Markham and Greenham 2021; Pascual et al. 2022). Additionally, heat waves are increasing in frequency and intensity (Mazdiyasni and AghaKouchak 2015; Alizadeh et al. 2020; Perkins‐Kirkpatrick and Lewis 2020; IPCC 2023), droughts are becoming more severe and spatially extensive (Vogel et al. 2019), and soil constraints, including salinity, nutrient imbalances, microplastics, and heavy metal contamination, are expanding into irrigation‐driven agriculture and soil mismanagement, as well as multiple natural ecosystems (Rillig et al. 2019, 2021, 2023).

**FIGURE 1:**
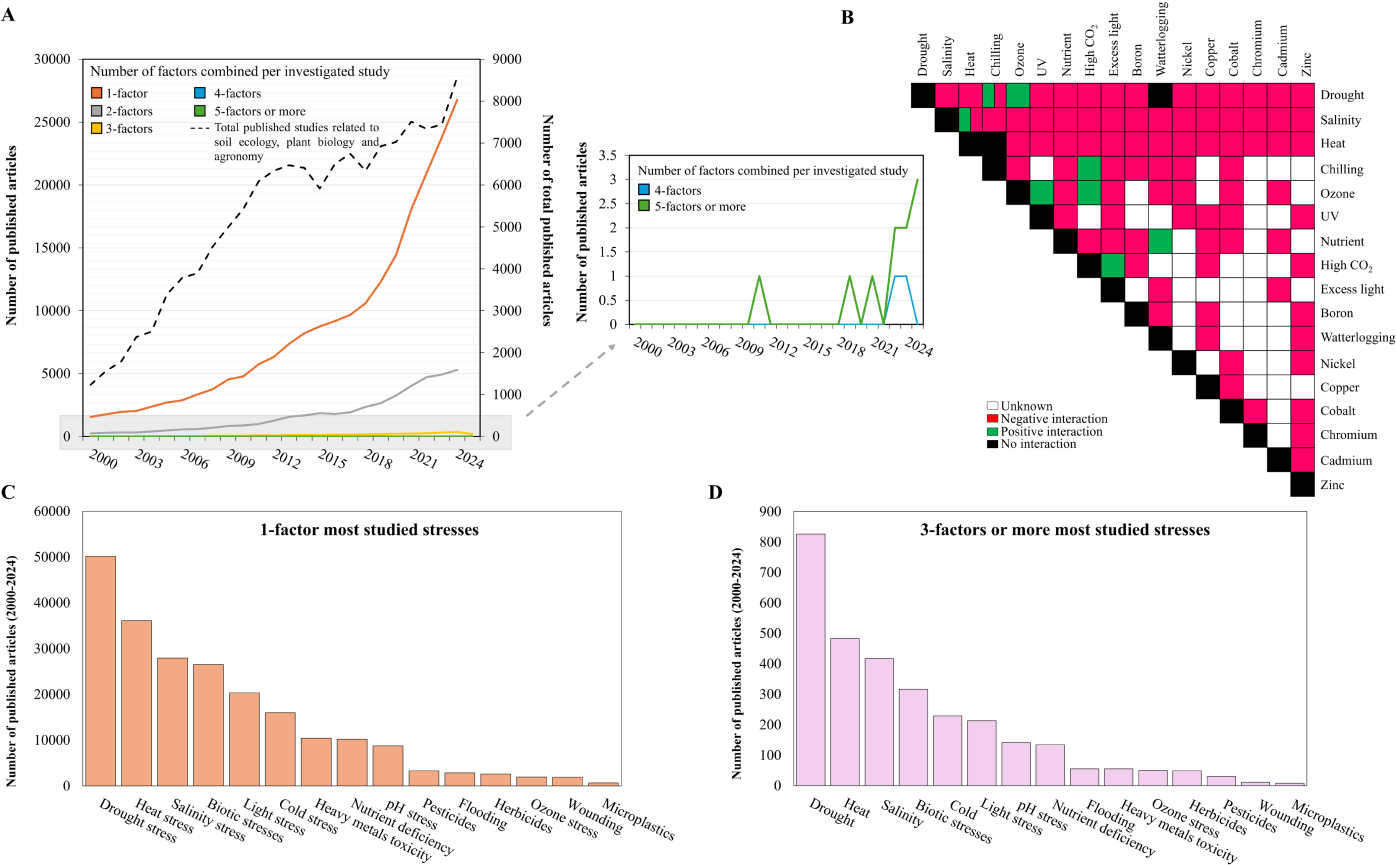
Trends in studies addressing abiotic stress in soil and plant systems. (A) Temporal progression in the number of published articles studying single or multiple stress factors, showing the relative scarcity of studies addressing multifactorial stress combination over the last two decades. (B) Interaction matrix summarizing reported relationships between pairs of abiotic stressors, with magenta, green, and black cells indicating putative negative, positive, and neutral (no) interactions, respectively; white cells correspond to unknown or unstudied interactions. *Source:* Adapted from Mittler (2006), Suzuki et al. (2014), and Zandalinas et al. (2016). (C) Ranking of the most studied single (1–factor) stresses. (D) Frequency of stresses investigated in studies combining three or more factors.

These stressors rarely occur in isolation. Instead, they overlap temporally and spatially, generating complex environmental stress conditions in which temperature, water availability, soil, water, and air chemistry, relative humidity, and light intensity and quality interact dynamically (Rivero et al. 2021; Zandalinas, Fritschi, and Mittler 2021; Pascual et al. 2022; Zandalinas et al. 2024). As a result, plants are increasingly exposed to multiple interacting abiotic stresses that increase in their complexity. This convergence of stressors introduces a new dimension to plant stress biology. Here, we define high level of environmental stress complexity as three or more simultaneous or sequential occurring stress factors that interact, i.e., multifactorial stress combination (MFSC), and propose that high stress complexity acts as a systems‐level driver capable of reshaping the interactions and hierarchies between different individual stress‐response pathways co‐activated during MFSC, as well as triggering new MFSC‐specific regulators, rather than simply intensifying established individual stress‐response pathways co‐triggered during MFSC (Rillig et al. 2019, 2021, 2023; Bi et al. 2024; Sinha, Zandalinas, Peláez‐Vico, et al. 2025; Mittler et al. 2026). Despite growing environmental complexity, plant stress biology has largely relied on reductionist approaches, focusing on responses to individual stresses such as drought, salinity, heat, cold, or excess light (Mittler 2006; Suzuki et al. 2014; Zhu 2016; Sinha, Zandalinas, Peláez‐Vico, et al. 2025). Consistent with this trend, a quantitative survey of publications across soil ecology, plant biology and agronomy, from 2000 to 2024, revealed a notable gap between real‐world conditions and experimental designs (Figure 1A). Thus, most studies still examine individual stress factors, with only a substantial fraction addressing two‐factor combinations, whereas investigations incorporating three or more simultaneous stresses remain relatively rare (Figure 1A). Importantly, this imbalance persists despite the steady increase in overall stress‐related research outputs over the past two decades, indicating that the expansion in research papers in the field has not been accompanied by a comparable shift towards higher‐order stress integration.

The consequences of this limitation become evident when considering the diversity of interactions reported among abiotic stressors. The interaction matrix summarizing the impact of different combinations of two‐stress factors (Figure 1B; originally introduced by Mittler 2006 and later updated by Zandalinas et al. 2016) illustrates that stress interactions can vary in their outcome. Reported interactions are frequently negative, but positive interactions also occur (Rasmussen et al. 2013; Rivero et al. 2021; Shaar‐Moshe et al. 2017; Zhang and Sonnewald 2017), demonstrating that combined stresses can either exacerbate or partially buffer individual effects. Crucially, responses to combined stresses are rarely additive and often generate distinct physiological and transcriptional states (Suzuki et al. 2013; Balfagón et al. 2024; Sinha, Zandalinas, Peláez‐Vico, et al. 2025; Mittler et al. 2026).

**FIGURE 2:**
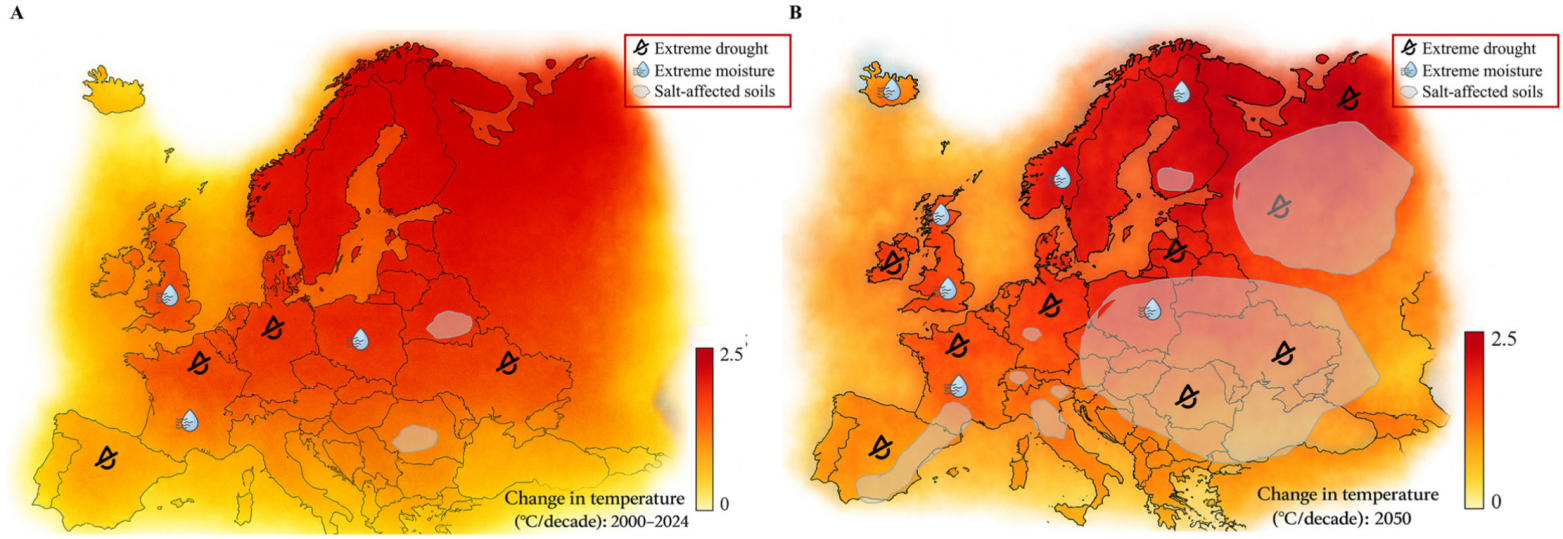
Changes in temperature, drought, and salinity stressors across Europe. Maps illustrating the spatial distribution of mean surface temperature change (°C per decade) during two distinct periods: From 2000 to 2024 (A) and projected from 2025 to 2050 (B). The color gradient represents the rate of temperature increase, ranging from 0°C/decade to 2.5°C/decade. Symbols indicate regions under major abiotic stress conditions: Extreme drought (black symbol), extreme moisture (light blue symbol), and salt‐affected soils (gray shaded areas). Data sources: Copernicus Climate Data Store (CDS, sea and earth's surface temperature and soil moisture; https://cds.climate.copernicus.eu); European Drought Observatory (EDO, drought indices); European Soil Data Centre (ESDAC, salt‐affected soils).

Research on combined stresses has concentrated on a subset of environmental factors, shaping our current understanding of plant responses to stress combinations. Single‐factor studies are concentrated on common stress factors, particularly drought, heat, and salinity (Figure 1C). Likewise, among studies combining three or more factors, research effort is mainly centered on drought‐ or water deficit‐driven scenarios, whereas many plausible multifactorial contexts, such as heavy metal exposure, nutrient imbalance, UV radiation or waterlogging, are underrepresented (Figure 1D). These patterns reveal a gap between natural complexity and research focus: while multiple environmental factors occur together in nature, experiments mostly examine one or two stresses, with certain factors receiving more attention than others (Figure 1). Consequently, the regulatory configurations that emerge under higher‐order stress integration remain poorly resolved. The implications of this gap become more evident when current experimental trends (Figure 1) are viewed alongside projected climatic convergence (Figure 2). Spatial analyses of Europe show not only progressive warming between 2000 and 2024 and projected worsening of these conditions for 2025–2050, but also an expanding geographic overlap among extreme drought, increased moisture regimes and salinity‐affected soils. For example, parts of Central and Eastern Europe are projected to experience increasing overlap between drought and salt‐affected soils, while Mediterranean regions show recurrent drought signals coinciding with expanding salinity and heat risks. In contrast, northern Europe and Scandinavia display increasing associations between warming and extreme moisture regimes (Figure 2; data sources: Copernicus Climate Data Store [CDS, sea and earth's surface temperature and soil moisture; https://cds.climate.copernicus.eu]; European Drought Observatory [EDO, drought indices]; European Soil Data Centre [ESDAC, salt‐affected soils]). These stresses increasingly co‐occur within the same regions, shifting landscapes from predominantly single‐driver regimes towards multi‐driver configurations. Multifactorial stress environments are, therefore, likely to become structurally embedded within agroecosystems rather than being episodic anomalies (Pascual et al. 2022; Zandalinas et al. 2024).

The following sections examine how increasing stress complexity reshapes plant hormonal and physiological regulation across interconnected biological scales. Rather than simply amplifying well‐established stress‐response pathways, the evidence suggests that these conditions trigger context‐dependent reorganization of stress‐response regulatory architecture. Because the available studies span diverse species, developmental stages, and experimental systems, the patterns discussed here should be viewed as emerging trajectories of stress‐response regulatory integration under MFSC, rather than universally conserved responses.

### 1.2 Hormonal Networks Under MFSC

Increasing environmental complexity raises a central mechanistic question: how do plants integrate multiple simultaneous abiotic inputs via their regulatory networks? Hormonal signaling pathways constitute one of the principal integrative layers linking environmental perception to transcriptional reprogramming, metabolic adjustment and developmental prioritization (Mittler 2006; Yoshida et al. 2010, 2014; Suzuki et al. 2014; Zhu 2016; Hsu et al. 2021; Fàbregas et al. 2026). Under single‐stress scenarios, relatively defined hormonal signatures are often associated with specific environmental cues, including abscisic acid (ABA) with drought and salinity, jasmonic acid (JA) with wounding and defense, salicylic acid (SA) with heat stress and biotic responses, and ethylene with stress acclimation and growth modulation (Vlot et al. 2009; Cutler et al. 2010; Wasternack and Hause 2013; Kazan 2015; Dubois et al. 2018). Although extensive crosstalk exists among hormonal pathways, the general frameworks described above for single stressors allow the identification of dominant hormonal axes.

**FIGURE 3:**
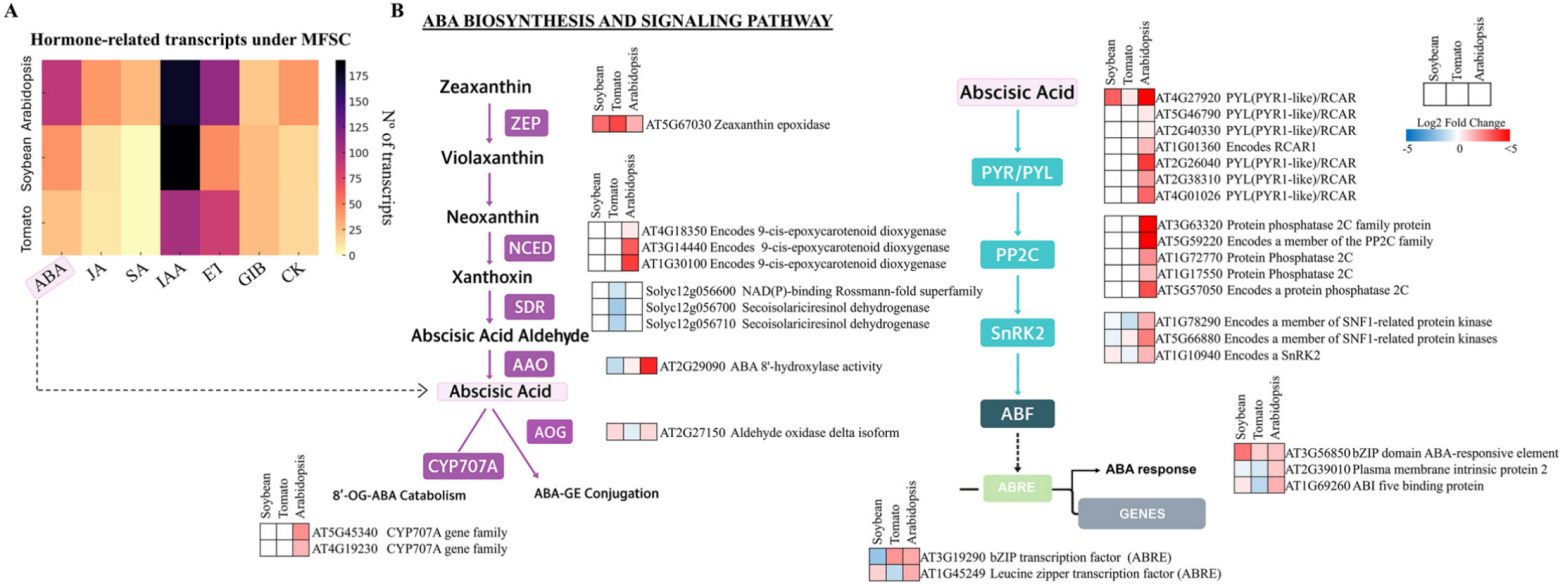
Hormonal regulation under multifactorial stress conditions across representative species. (A) Heatmap showing the number of hormone‐associated transcripts identified in *Arabidopsis thaliana* , soybean, and tomato, under multifactorial stress combination (MFSC; *Source:* Data adapted from Zandalinas, Sengupta, et al. (2021), Peláez‐Vico et al. (2024), Pascual, Serna, et al. (2025)). (B) Overview of abscisic acid (ABA) biosynthesis and signaling transcript expression during multifactorial stress combination across species, highlighting conserved and species‐specific transcriptional responses to MFSC. Abbreviations: AAO, abscisic aldehyde oxidase; ABA, abscisic acid; ABF, ABA‐responsive element‐binding factor; ABRE, ABA‐responsive element. AOG, ABA glucose ester‐forming enzyme; CK, cytokinin; CYP707A, ABA 8′‐hydroxylase; ET, ethylene; GIB, gibberellin; IAA, indole‐3‐acetic acid; JA, jasmonic acid; NCED, 9‐cis‐epoxycarotenoid dioxygenase; PP2C, protein phosphatase 2C; PYR/PYL, pyrabactin resistance/pyrabactin resistance‐like receptor; SA, salicylic acid; SDR, short‐chain dehydrogenase/reductase; SnRK2, SNF1‐related protein kinase 2; ZEP, zeaxanthin epoxidase.

In contrast, MFSC fundamentally challenges this basic architecture (Zhang and Sonnewald 2017; Pascual et al. 2023; Alptekin and Burritt 2025; Pascual, Mohanty, et al. 2025; Pascual, Serna, et al. 2025; Jiang et al. 2025). Comparative analyses across Arabidopsis, soybean and tomato revealed broad but non‐uniform reprogramming of hormone‐related transcripts under MFSC (Figure 3A). A consistent feature across species is enrichment of ABA‐associated transcripts, underscoring the conserved role of ABA as a central integrator of osmotic and ionic components, frequently embedded in stress combination scenarios (Zhu 2016; Devireddy et al. 2020). However, as shown in Figure 3A, substantial representation of auxin (IAA)‐related transcripts is found during MFSC, particularly in Arabidopsis and soybean, indicating that MFSC engages growth‐regulatory modules. This co‐enrichment suggests that MFSC triggers regulatory rebalancing between growth maintenance and stress prioritization, rather than simple amplification of stress‐associated hormones. JA‐, SA‐ and ethylene‐related transcripts are present but comparatively variable across species (Figure 3A, upper panel), reinforcing the interpretation that hormonal responses under MFSC are combinatorial and context‐dependent rather than dominated by a single pathway (Pascual et al. 2023; Pascual, Serna, et al. 2025).

Detailed analysis of the ABA pathway in soybean, tomato, and Arabidopsis, indicates that redistribution in transcript expression occurs within this pathway architecture (Figure 3B). MFSC‐associated modulation spans changes in the expression of biosynthetic enzymes, i.e., 9‐cis‐epoxycarotenoid dioxygenase (NCED), zeaxanthin epoxidase (ZEP) or abscisic aldehyde oxidase (AAO), abscisic acid receptors (pyrabactin resistance/pyrabactin resistance‐like) (PYR/PYL), protein phosphatases type 2C (PP2C), sucrose non‐fermenting 1‐related protein kinases 2 (SnRK2), and downstream transcriptional regulators such as ABA‐responsive element‐binding factors/ABA‐responsive elements (ABF/ABRE) modules (Figure 3B). Importantly, the relative distribution of pathway modulation across these nodes differs among species. In Arabidopsis, MFSC prominently affects biosynthetic and downstream response components (Figure 3B; Zandalinas, Sengupta, et al. 2021), whereas in soybean and tomato transcript changes are more widely distributed across signaling modules, including PYR/PYL receptors and PP2C/SnRK2 transcripts (Figure 3B; Peláez‐Vico et al. 2024; Pascual, Serna, et al. 2025). Such variation suggests that MFSC does not uniformly intensify ABA output but may instead alter the balance between hormone biosynthesis and signaling sensitivity. Given that receptor‐phosphatase‐kinase modules determine response sensitivity, feedback dynamics and temporal stability (Cutler et al. 2010), these species‐specific patterns imply differential tuning of ABA responsiveness under environments with increased complexity.

Integration of ABA cascade transcriptomics and proteomics data (Sinha et al. 2024; Pascual, Mohanty, et al. 2025; Pascual, Serna, et al. 2025) reveals an additional layer of regulation. Thus, protein level changes across biosynthetic and signaling modules do not always parallel transcript level patterns, indicating partial decoupling between transcriptional activity and protein accumulation (Sinha et al. 2024; Pascual, Mohanty, et al. 2025; Pascual, Serna, et al. 2025). Notably, modulation extends across the entire ABA pathway, from carotenoid precursors to ABRE‐associated transcriptional outputs, rather than concentrating at a single control point. Such distributed regulation is consistent with multi‐omics analyses showing that complex stress environments induce buffering and reweighting across regulatory layers (Sinha et al. 2024; Pascual, Mohanty, et al. 2025; Pascual, Serna, et al. 2025). The patterns shown in Figure 3 indicate that increasing the number of simultaneous stresses does not simply intensify a single stress pathway, instead, it appears to reorganize hormonal signaling networks, influencing processes ranging from hormone biosynthesis and perception to downstream transcriptional regulation. Such redistribution of hormonal control is likely to propagate beyond transcriptional regulation, re‐shaping the physiological coordination of plant responses under increased stress complexity. In future studies it would be important to quantify the levels of different plant hormones in vegetative and reproductive tissues of different plants and crops under conditions of MFSC (Peláez‐Vico et al. 2024).

While the patterns identified across hormonal, physiological, and transcriptional layers under MFSC are mostly consistent across multiple studies, the mechanistic basis underlying these responses remains understudied. For example, the enrichment of ABA‐associated transcripts across species suggests a central integrative role for ABA under MFSC. However, whether ABA acts as a primary driver of the observed physiological and transcriptional reprogramming, or instead reflects a downstream consequence of stress integration, remains to be experimentally established. Current data support the existence of non‐additive and system‐level responses under MFSC, but the causal architecture linking stress perception, hormonal signaling, and downstream physiological and transcriptional outcomes remains to be fully elucidated. Addressing these questions will require targeted experimental approaches. For instance, the use of hormone‐deficient or signaling mutants (e.g., ABA biosynthesis or signaling mutants) under controlled MFSC conditions could help determine the causal contribution of specific hormonal pathways. Likewise, perturbation of key regulatory nodes within signaling networks, combined with multi‐omics and physiological measurements, could clarify whether threshold‐like responses emerge from network regulatory hubs, feedback regulation, or resource limitation.

### 1.3 Physiological Responses Under MFSC

The broad hormonal reconfiguration observed under MFSC is not confined to transcriptional domains but propagates into coordinated physiological reprograming at the whole‐plant level. Hormonal pathways, particularly ABA‐dependent signaling, directly regulate guard cell dynamics, transpiration and carbon assimilation, thereby coupling environmental perception to gas exchange performance (Buckley 2019; Lawson and Matthews 2020). Previous work on combined drought‐heat and drought‐salinity stresses has shown that these interactions result in stomatal closure and strong reductions in photosynthesis relative to control or single stresses (Rizhsky et al. 2002, 2004; Mittler 2006; Suzuki et al. 2014; Devireddy et al. 2020). These studies suggest that stress integration alters how physiological processes are coordinated rather than simply amplifying individual responses. As hormonal hierarchies are redistributed under increasing stress complexity (Figure 3), these regulatory shifts are likely to be reflected in changes in the whole‐plant physiological state.

**FIGURE 4:**
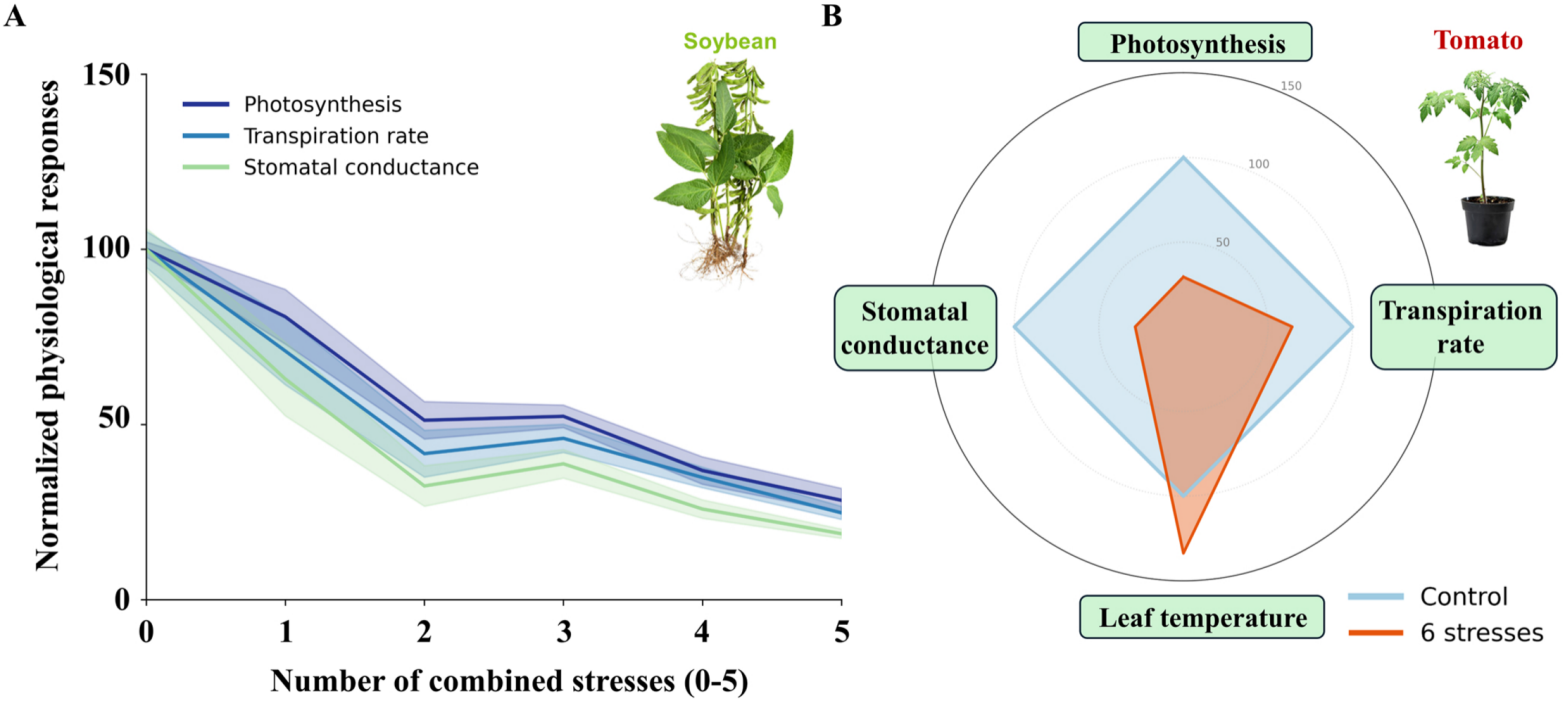
Physiological responses to multifactorial stress conditions. (A) Decrease in photosynthesis, transpiration rate, and stomatal conductance, as the number of combined stresses increases in soybean during multifactorial stress combination (MFSC). *Source:* Data adapted from Peláez‐Vico et al. (2024). Shaded areas represent the standard deviation among replicates. (B) Radar plot comparing tomato physiological responses to MFSC (orange), compared to control plants (blue; normalized to 100%). *Source:*Data adapted from Pascual et al. (2023).

This pattern is particularly evident in soybean (Figure 4A). As the number of simultaneously imposed stresses increases from one to five, net photosynthesis, transpiration rate, and stomatal conductance decline progressively and in parallel (Peláez‐Vico et al. 2024). This trend suggests that conserving water becomes a central regulatory priority as stress complexity increases (Impa et al. 2019; Santiago and Sharkey 2019; Sinha et al. 2022; Jensen et al. 2024; Sinha, Peláez‐Vico, Pascual, et al. 2025). The enrichment of ABA‐associated transcripts under MFSC (Figure 3) provides a potential mechanistic basis for this coordination, consistent with established roles of ABA in promoting stomatal closure under osmotic and water deficit conditions (Suzuki et al. 2016; Devireddy et al. 2020). The steady increase in stomatal closure with the increasing number of stress factors impacting the plant indicates that complexity itself functions as a regulatory variable, even when each individual stress is applied at moderate intensity. Similarly, tomato plants exposed to six simultaneous stresses show a comparable reduction across different physiological processes (Figure 4B). Photosynthesis, transpiration, and stomatal conductance decline concurrently, relative to control plants, under the combination of six stresses (salinity, heat stress, excess light, oxidative stress induced by paraquat, nitrogen deficiency and cadmium toxicity), while leaf temperature increases (Pascual et al. 2023). Elevated leaf temperature reflects diminished transpirational cooling capacity, indicating that stomatal restriction reduces thermal dissipation under MFSC (Figure 4B).

The coordinated decline in transpiration and carbon assimilation therefore reflects a redefinition of physiological tradeoffs. For example, under heat stress, partial stomatal opening can sustain evaporative cooling and preserve photosynthetic integrity (Rizhsky et al. 2002, 2004; Wahid et al. 2007). Under MFSC that includes osmotic or ionic components, stomatal restriction dominates even in the presence of thermal stress (Figure 4A,B), indicating prioritization of hydraulic safety over cooling efficiency. Comparable prioritization has been observed under drought‐salinity combinations, where carbon assimilation is reduced to maintain water status despite metabolic costs (Suzuki et al. 2014; Devireddy et al. 2020). Increasing stress complexity therefore restricts the range of physiological responses available to the plant, shifting it towards conditions characterized by reduced carbon gain, limited transpiration, and potentially causing a greater heat stress (Zandalinas and Mittler 2022; Sage 2020).

Interestingly, regulation of transpiration under stress combinations is not limited to uniform whole‐plant suppression. Recent work on stress combinations demonstrated that they can restructure water flux in a spatially selective manner. Sinha, Peláez‐Vico, Fritschi, and Mittler (2025) and Sinha, Peláez‐Vico, Pascual, et al. (2025) described a process termed ‟differential transpiration” that occurred under a combination of heat and drought, where stomatal closure is evident in vegetative tissues but not in reproductive organs. This organ‐specific partitioning enables localized cooling of heat‐sensitive reproductive structures despite systemic water limitation, effectively decoupling leaf‐level hydraulic conservation from reproductive thermoregulation. These findings illustrate how plants can selectively redistribute water flux among organs under combined stress conditions. In this context, MFSC may also cause the reorganization of hydraulic prioritization across tissues, rather than simply reducing transpiration at the whole‐plant level. Thus, system‐wide reductions in physiological performance and shifts in hydraulic allocation could be separated: while overall gas‐exchange capacity declines (Figure 4A,B), water flux could be selectively maintained in tissues critical for reproductive success (i.e., differential transpiration). Such a pattern could indicate that increasing stress complexity might reshape physiological coordination through shifts in regulatory priorities rather than through uniform suppression of plant function. Future studies of stomatal regulation, as well as other physiological responses of vegetative and reproductive tissues of different plant species, subjected to MFSC, are needed to address these possibilities.

### 1.4 Transcriptional Responses of Photosynthesis‐Related Pathways Under MFSC

**FIGURE 5:**
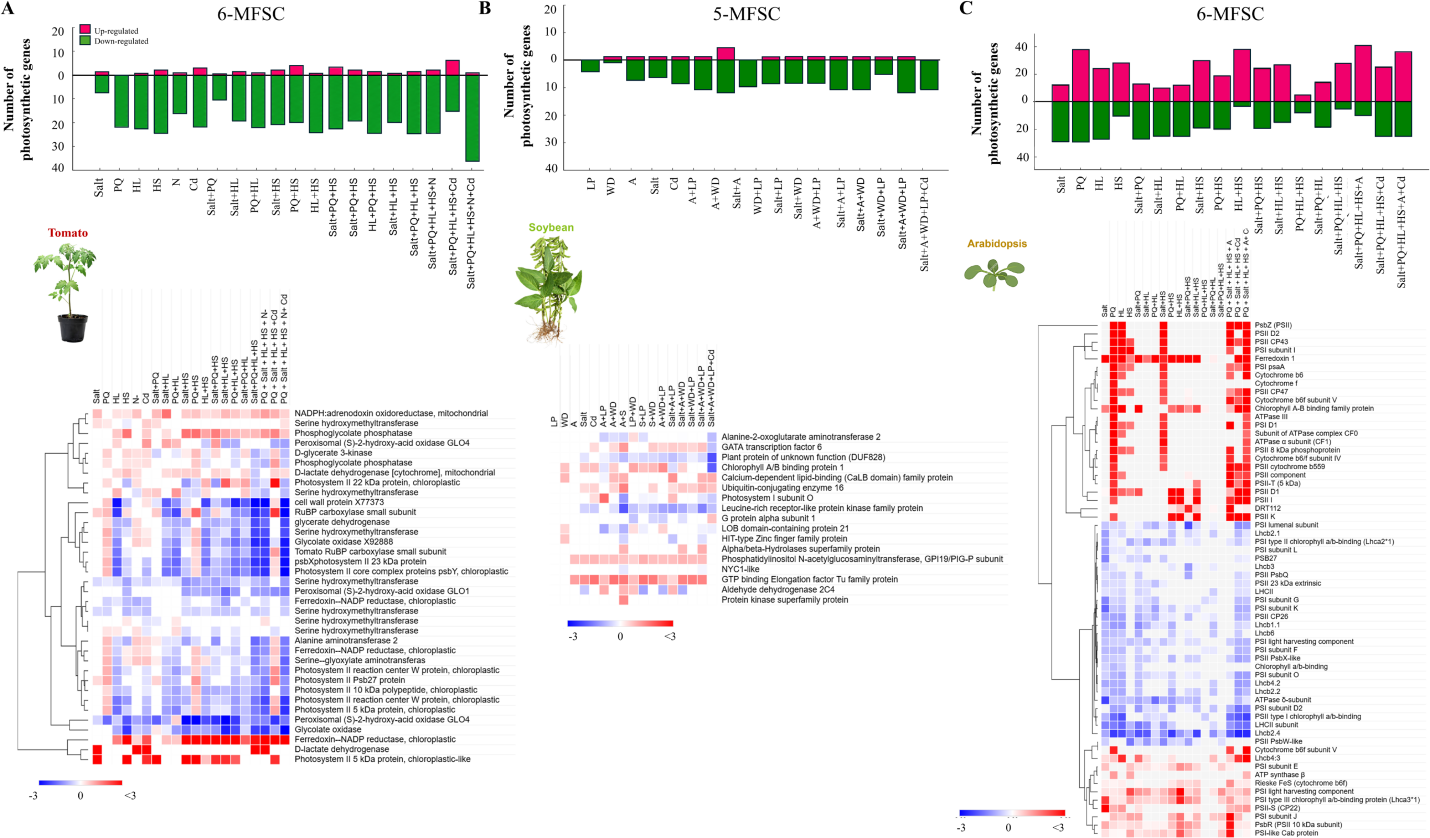
Regulation of photosynthesis–related gene expression in tomato, soybean, and *Arabidopsis thaliana* subjected to multifactorial stress conditions. (A) Bar graphs showing the number of photosynthesis‐associated transcripts upregulated (magenta) and downregulated (green) in tomato under single and combined stress conditions, including salt (S), paraquat (PQ), excess light (HL), heat stress (HS), heavy metal (Cd) and N deficiency (N) as well as their multifactorial combinations. The heatmap below shows hierarchical clustering of differentially expressed photosynthetic transcripts (log_2_ fold change) across the same stress treatments. (B) Same as in A, but for transcriptional responses in soybean under single and combined stress conditions, including salt (S), acidity (A), water deficit (WD), heavy metal (Cd) and low phosphate (LP) as well as their multifactorial combinations (C) Same as A, but for transcriptional response in Arabidopsis under single and combined stress conditions, including salt (S), paraquat (PQ), excess light (HL), heat stress (HS), heavy metal (Cd) and acidity (A) as well as their multifactorial combinations. *Source:* Data was obtained from Zandalinas, Sengupta, et al. (2021), Peláez‐Vico et al. (2024), and Pascual, Serna, et al. (2025). Abbreviations: A, acidity; Cd, cadmium toxicity; HL, high (excess) light; HS, heat stress; LP, low phosphate; N, nitrogen deficiency; PQ, paraquat; WD, water deficit.

The physiological changes described above suggest that sustained reductions in gas exchange under MFSC, due to stomatal closure, could result in drastic reductions in photosynthesis. However, persistent declines in photosynthesis (Figure 4) could also result from changes in nuclei and chloroplast gene expression. Such changes become evident when examining transcriptional responses of photosynthesis‐associated genes under MFSC, especially in tomato (Figure 5; Zandalinas, Sengupta, et al. 2021; Peláez‐Vico et al. 2024; Pascual, Serna, et al. 2025).

Across tomato, soybean and Arabidopsis, increasing stress complexity is associated with widespread transcriptional reprogramming of photosynthesis‐related genes (Figure 5, upper panels). However, this pattern is not equally pronounced across species. In tomato and soybean, higher‐order stress combinations lead to a clear predominance of downregulated photosynthesis‐associated transcripts, with the balance between up‐ and down‐regulated genes progressively shifting towards repression as additional stressors are combined (Figure 5; Zandalinas, Sengupta, et al. 2021; Peláez‐Vico et al. 2024; Pascual, Serna, et al. 2025). In contrast, in Arabidopsis this trend is less evident, with a more heterogeneous distribution of transcriptional responses under comparable conditions (Figure 5). The genes predominantly affected in tomato and soybean include components of photosystem I and II, light‐harvesting complexes and Calvin cycle enzymes, which are central to light capture, electron transport and carbon fixation (Nelson and Yocum 2006; Rochaix 2011; Foyer et al. 2012). The coordinated repression of these modules suggests a reduction in photosynthetic capacity under increasing stress complexity, likely contributing to the maintenance of cellular redox balance and the prevention of photo‐oxidative damage when carbon assimilation becomes constrained (Apel and Hirt 2004; Foyer and Noctor 2005; Miller et al. 2008; Tikkanen and Aro 2014; Sewelam et al. 2016). Importantly, hierarchical clustering reveals that MFSC forms distinct transcriptional clusters rather than represents linear extensions of single‐stress profiles, indicating that MFSC induces emergent transcriptional states within the photosynthetic network (Shaar‐Moshe et al. 2017; Ashoub et al. 2018; Nguyen et al. 2025).

In tomato, repression encompasses genes encoding photosystem I and II (PSI and PSII, respectively) subunits, light harvesting complex proteins, ferredoxin‐NADP reductases and Calvin cycle enzymes (Figure 5A; Pascual, Serna, et al. 2025). The coordinated clustering of these genes indicates a global reduction of chloroplast activity rather than isolated pathway inhibition. Similarly, soybean under MFSC shows a parallel predominance of downregulated photosynthetic transcripts (Figure 5B; Peláez‐Vico et al. 2024), indicating convergence towards reduced investment in excitation capture, electron transport and carbon fixation. In contrast, Arabidopsis displays a more heterogeneous transcriptional response, with both up‐ and down‐regulated photosynthesis‐related genes across conditions (Figure 5C; Zandalinas, Sengupta, et al. 2021), suggesting a less coordinated repression of chloroplast functions under comparable stress combinations. Although the specific genes affected vary among species and the specific stresses applied, the functional outcome converges: MFSC reduces transcriptional investment in excitation capture, electron transport and carbon fixation.

Increasing complexity appears therefore to lower the threshold at which carbon assimilation becomes transcriptionally deprioritized. In tomato, repression extends simultaneously to Rubisco subunits, PSII core proteins (i.e., Psb family members), PSI components and chlorophyll‐binding proteins (Figure 5A), indicating concurrent limitations at both photochemical and biochemical levels, while in Arabidopsis, repression primarily occurs in antenna proteins and selected PS core proteins, while others are upregulated (Figure 5C). The diffusive limitations shown in Figure 4 are further reinforced by reduced chloroplast metabolic capacity, linking physiological reductions with changes at the molecular level (Pascual, Serna, et al. 2025). Mechanistically, coordinated repression of excitation processes and carbon fixation may help reduce excitation pressure and limit photo‐oxidative damage when carbon assimilation is restricted (Apel and Hirt 2004; Foyer and Noctor 2005; Miller et al. 2008; Tikkanen and Aro 2014; Sewelam et al. 2016; Chen et al. 2017). Under single stresses, plants often retain partial photosynthetic function while activating protective pathways (Mittler 2006; Suzuki et al. 2014). Under higher‐order combinations, sustained expression of light‐harvesting and electron transport machinery may become metabolically and redox‐wise untenable. Downregulation of these modules under MFSC therefore likely reflects active regulatory prioritization rather than passive deterioration.

Although transcriptional architectures differ among tomato, soybean, and Arabidopsis reflecting species‐specific chloroplast regulation and developmental context, the convergence towards photosynthetic repression is consistent (Zandalinas, Sengupta, et al. 2021; Sinha et al. 2024; Pascual, Serna, et al. 2025). As environmental complexity increases, plant metabolism shifts away from growth‐oriented carbon investment towards a more constrained metabolic state. Under MFSC, stomatal restriction reduces transpirational cooling (Figure 4) and is accompanied by repression of chloroplast‐related genes (Figure 5), collectively reinforcing a survival‐oriented physiological state.

At the mechanistic level, the different MFSC‐associated transcriptional patterns raise the possibility that photoprotective and electron transport processes are also reconfigured under MFSC. For instance, non‐photochemical quenching (NPQ), largely mediated by proteins such as PsbS, may play a key role in dissipating excess excitation energy, as heat. Given the reduction in transpirational cooling, suppression of NPQ expression could represent an additional layer of thermal and redox control under complex stress environments (Figures 4 and 5; Li et al. 2000; Niyogi 2000; Müller et al. 2001; Horton et al. 2008; Ruban 2016). Similarly, MFSC may alter the balance between PSII and PSI activity, potentially favoring cyclic electron flow around PSI to maintain ATP production while limiting over‐reduction of the electron transport chain. Although current transcriptomic datasets do not provide sufficient resolution to conclusively determine whether NPQ components or PSI/PSII antenna and core subunits are differentially prioritized, the coordinated repression of photosynthetic genes suggests that adjustments in energy dissipation and electron flow are likely integral to the reprogramming of chloroplast function under increasing stress complexity. Future studies integrating chlorophyll fluorescence, biochemical measurements and high‐resolution transcriptomic analyses will be required to resolve these mechanisms.

### 1.5 A Complexity‐Driven Regulatory Reconfiguration Model for MFSC

**FIGURE 6:**
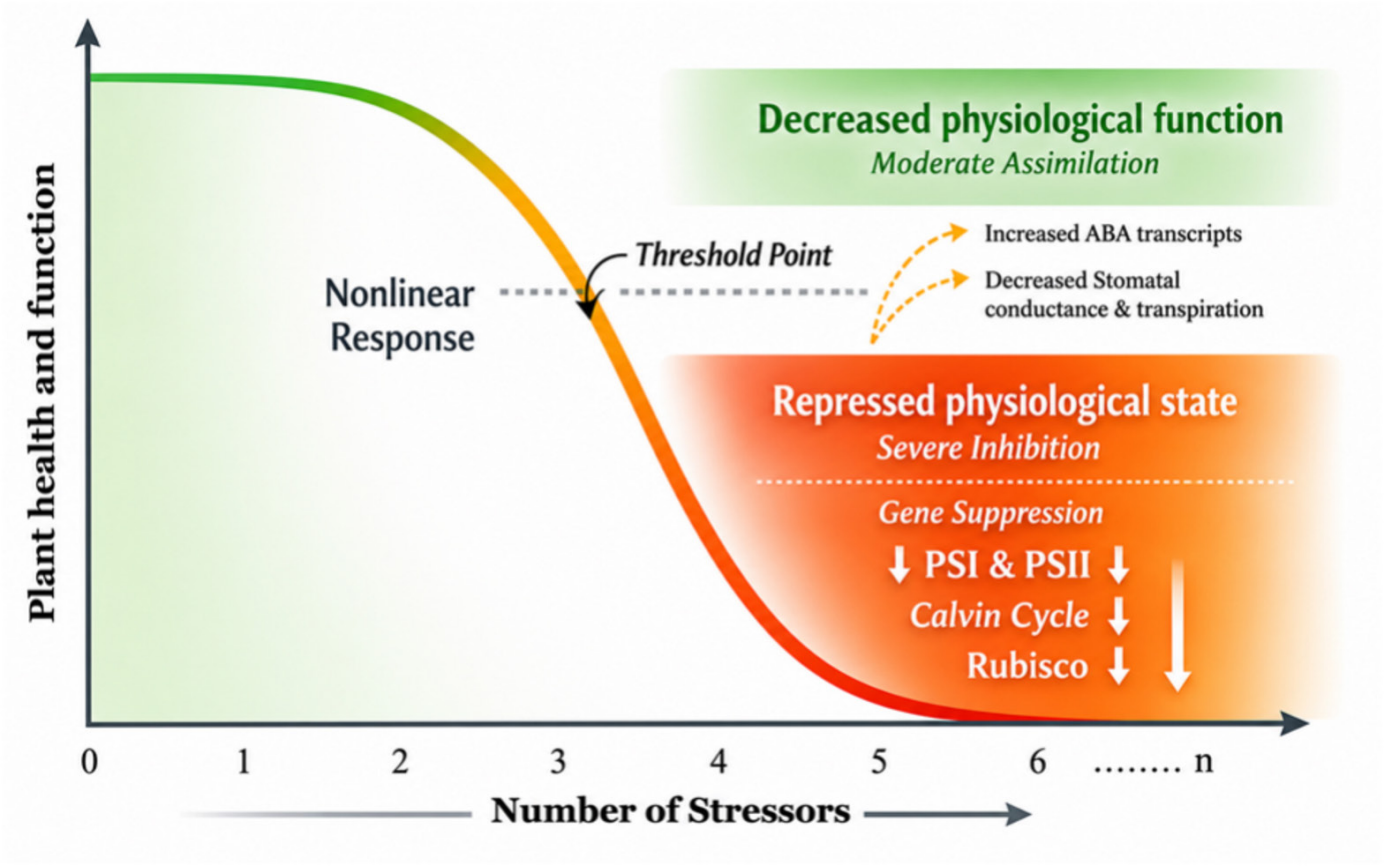
Proposed model of complexity–driven physiological and transcriptional reconfiguration under multifactorial stress combinations. As stress complexity increases (*X*‐axis; number of stressors from 1 to *n*), photosynthetic output follows a nonlinear trajectory (*Y*‐axis). Under low stress complexity (1–2 stressors), plants maintain partial physiological function and moderate carbon assimilation. As additional stressors accumulate (3–4 or more stressors, multifactorial stress combination; MFSC), regulatory integration approaches a threshold at which coordinated downscaling of gas exchange becomes evident. Beyond this threshold (≥ 3–4 stressors), MFSC induces a repressed physiological state characterized by diminished net photosynthesis and transcriptional suppression of photosystem I and II components, Calvin cycle enzymes and Rubisco, associated with increased ABA‐related transcripts and reduced stomatal conductance and transpiration.

The multi‐layered patterns described above (hormonal redistribution, systemic physiological reduction, and coordinated repression of photosynthetic‐associated genes) converge on a unifying principle: MFSC does not simply intensify individual stress responses but progressively reorganizes regulatory hierarchy as environmental complexity increases. We therefore propose a complexity‐driven regulatory reconfiguration model (Figure 6) in which rising combinatorial load induces state transitions that reflect crossing thresholds, rather than a linear decline in performance.

In the model shown in Figure 6, photosynthetic output declines along a nonlinear trajectory as the number of simultaneous stressors increases. Under low‐complexity conditions (one to two stressors), plants retain regulatory flexibility. Hormonal signaling is activated, yet gas exchange and carbon assimilation remain partially sustained. This corresponds to a “partial function” regime in which acclimatory plasticity compensates for moderate constraint, consistent with responses described under single or two stress combination (Mittler 2006; Rivero et al. 2021; Suzuki et al. 2014). As additional stressors accumulate (three or more; MFSC), regulatory control becomes redistributed within hormonal, metabolic, and other signaling networks. For example, ABA‐associated components are consistently engaged across species under MFSC (Zandalinas, Sengupta, et al. 2021; Peláez‐Vico et al. 2024; Pascual, Serna, et al. 2025). However, the specific points of regulation differ among species (biosynthetic activation in Arabidopsis, redistribution of signaling components in soybean, and shifts in receptor sensitivity in tomato; Figure 3). In our model, this redistribution reflects an approach to a regulatory threshold. Beyond this point, the system shifts towards a more repressed physiological state. Experimentally, this is accompanied by coordinated reductions in photosynthesis, transpiration, and stomatal conductance as stress combinations intensify (Pascual et al. 2023; Peláez‐Vico et al. 2024). As stress complexity increases, the range of physiological responses available to the plant becomes progressively narrower, favoring hydraulic conservation and redox stability over sustained carbon assimilation (Zandalinas and Mittler 2022; Sinha, Peláez‐Vico, Pascual, et al. 2025). This reduction in regulatory flexibility suggests limits to the plant's capacity to buffer multiple stresses, as the number of interacting perturbations increases (Kotak et al. 2007; Krasensky and Jonak 2012). Beyond species‐ and context‐ dependent levels of complexity, plants eventually enter a restricted configuration characterized by systemic limitation of carbon assimilation at both stomatal and chloroplastic levels (Shaar‐Moshe et al. 2017; Ashoub et al. 2018; Zandalinas and Mittler 2022). The decline in photosynthetic output is, therefore, not purely cumulative damage but rather a manifestation of constrained regulatory solution space.

At higher levels of complexity, this shift consolidates transcriptionally. In many species, MFSC is associated with a dominant repression of photosystem components, light‐harvesting complexes and Calvin cycle enzymes (Figure 5; Zandalinas, Sengupta, et al. 2021; Pascual, Serna, et al. 2025; Peláez‐Vico et al. 2024). The magnitude of repression exceeds that observed under single stresses, indicating threshold‐dependent reorganization rather than proportional scaling. Downregulation of excitation and carbon fixation modules likely reduce photo‐oxidative risk (Mittler 2002; Apel and Hirt 2004; Foyer and Noctor 2005; Tikkanen and Aro 2014) but also signals commitment to a survival‐oriented metabolic configuration in which growth investment is systematically deprioritized. Importantly, the model does not imply a universal MFSC transcriptome. While the functional outcome (i.e., reduced photosynthetic capacity under high‐complexity stress) is conserved, the molecular pathways by which species reach this state could differ (Sinha et al. 2024; Pascual, Serna, et al. 2025). Interestingly, in almost all plant systems studied to date, the threshold in which MFSC has the highest effect on plants occurred at about the four‐stress combination level, indicating that at this level of complexity the plant enters a state in which survival pathways are prioritized above all other pathways (Zandalinas, Sengupta, et al. 2021; Zandalinas and Mittler 2022; Pascual et al. 2023; Peláez‐Vico et al. 2024; Sinha et al. 2024; Pascual, Serna, et al. 2025).

Environmental complexity may therefore act as a constraint that channels different regulatory architectures towards a limited set of stable physiological states. In systems terms, increasing stress complexity may shift plants between alternative regulatory states, ranging from flexible acclimation under low complexity to survival‐oriented configurations under higher‐order stress. From this standpoint, increasing environmental complexity alters not only the intensity of stress responses but also the organization of regulatory networks. MFSC therefore represents a qualitative shift in how plants integrate environmental information, moving from flexible acclimation towards survival‐oriented regulation as stress complexity increases.

## 2 Final Remarks: Towards a Complexity‐Centered Framework for Plant Stress Biology

Plant stress biology has long been centered around the reductionist concept: one stress, one dominant pathway, one adaptive program. Yet the environmental reality plants increasingly face is not singular but multidimensional. MFSC challenges the assumption that stress responses can be understood as simple extensions of single‐stress paradigms. The evidence synthesized in this review suggests that increasing stress complexity reorganizes regulatory hierarchies rather than merely intensifying existing pathways. As complexity increases, regulatory systems redistribute control: hormonal regulation shifts, the range of physiological responses narrows, and transcriptional support for photosynthesis becomes constrained.

Rethinking stress biology indeed requires redefining resilience. From an ecological perspective, environmental convergence represents an increase in system complexity, where interacting drivers reshape stability landscapes rather than acting independently (Rillig et al. 2019, 2021, 2023). Plant stress responses could also be viewed as the outcome of coordinated regulatory processes operating under MFSC. In this sense, plant responses to multifactorial stress reflect broader principles governing how complex biological systems respond to increasing environmental complexity. Under future climate scenarios (Figure 2B), the key trait may not be maximal tolerance to individual stresses, but the capacity to maintain regulatory stability as stress complexity increases. Likewise, predictive crop models should move beyond additive representations of stress and incorporate stress complexity as a key driver of nonlinear responses. In a world where multiple stresses increasingly occur together, the central question is no longer how plants respond to individual stresses, but how they integrate and prioritize their responses to these stresses during MFSC. Understanding how plants respond to MFSC (e.g., defining the flexibility, plasticity, thresholds, and evolutionary limits of this response) may ultimately determine our capacity to sustain crop productivity under accelerating environmental changes (Lobell et al. 2011; Zhao et al. 2017; Bailey‐Serres et al. 2019; Hein et al. 2021; Zandalinas and Mittler 2022). We therefore argue that stress complexity should be treated as a primary biological variable rather than a secondary experimental barrier. In a world where stresses rarely occur in isolation, the future of plant resilience may depend on how far regulatory systems can reorganize before flexibility gives way to constraint. Future experimental designs should explicitly incorporate increasing levels of stress complexity to capture these nonlinear regulatory transitions.

An important next step is to translate the concept of MFSC into predictive and applied frameworks relevant to crop improvement and ecosystem management. From a modeling perspective, current crop and ecosystem models typically represent stress effects as additive or independent factors. Incorporating MFSC as a variable reflecting stress number, interaction strength, and temporal overlap could improve the ability of these models to capture nonlinear and threshold‐like responses observed under realistic environmental conditions. From a breeding standpoint, recognizing stress complexity as a primary driver suggests that selecting for tolerance to individual stresses may be insufficient under future climate scenarios. Instead, breeding strategies could prioritize traits associated with regulatory stability and network robustness under increasing stress complexity, such as the capacity to maintain coordinated hormonal signaling, hydraulic balance, and controlled downregulation of photosynthesis under multifactorial conditions. In terms of experimental design, the MFSC framework highlights the importance of moving beyond single‐ and 2‐stress experiments towards systematically increasing levels of stress complexity. Designing experiments that progressively incorporate additional stressors could help identify thresholds, emergent properties, and critical regulatory nodes. Such approaches would also facilitate the generation of datasets suitable for developing and validating predictive models of plant performance under complex environmental scenarios. Integrating MFSC into modeling, breeding, and experimental frameworks provides therefore a pathway for translating conceptual advances into practical strategies for improving plant resilience under increasingly complex environmental conditions.

## Author Contributions

L.S.P., R.M.R., S.I.Z., and R.M. have performed conceptualization, data curation, formal analysis, methodology, writing and/or provided funding acquisition.

## Funding

This work was supported by National Science Foundation (grant numbers IOS‐2414183; IOS‐2110017, IOS‐2343815).

## Conflicts of Interest

The authors declare no conflicts of interest.

## Data Availability

Data sharing is not applicable to this article as all newly created data is already contained within this article.
